# Dexamethasone implant vs. anti-VEGF for diabetic macular edema with epiretinal membrane: short-term outcomes

**DOI:** 10.1186/s40942-025-00788-w

**Published:** 2026-01-02

**Authors:** Yung-Ching Chang, Tsung-Cheng Hsieh, Tsung-Yueh Chan, Ming-Shan He

**Affiliations:** 1 Department of Ophthalmology, Buddhist Tzu Chi General Hospital, Tzu Chi University, No. 707, Sec. 3 Chung-Yung Road, Hualien, 970 Taiwan; 2 Institute of Medical Sciences, Tzu Chi University, Hualien, Taiwan; 3 Department of Ophthalmology and Visual Science, Tzu Chi University, Hualien, Taiwan; 4 Department of Ophthalmology, Mennonite Christian Hospital, Hualien, Taiwan

**Keywords:** Anti-VEGF, Diabetic macular edema, Dexamethasone implant, Epiretinal membrane

## Abstract

**Background:**

Diabetic retinopathy frequently coexists with secondary epiretinal membrane (ERM), which may interfere with therapeutic responses in diabetic macular edema (DME). However, consensus on optimal management for DME with concomitant ERM is lacking. This study compared the short-term therapeutic effects of intravitreal dexamethasone (DEX) implants and anti-VEGF agents in DME eyes with ERM.

**Methods:**

We retrospectively analyzed 109 eyes of 71 patients treated with either intravitreal anti-VEGF or DEX implant for DME between January 2014 and December 2022. Pre-treatment optical coherence tomography was reviewed. Best-corrected visual acuity (BCVA) and central macular thickness (CMT) were recorded at baseline, 2 months, and 4 months after treatment. The primary outcomes were changes in BCVA and CMT between baseline and follow-up after DEX or anti-VEGF therapy.

**Results:**

DEX provided a significantly greater relative reduction in CMT for DME eyes with ERM (β = − 15.675% [5.0]), while no significant difference was observed in eyes without ERM compared to anti-VEGF. Anti-VEGF therapy resulted in significantly less CMT reduction in eyes with ERM than in those without ERM (*P* < 0.001). DEX implantation led to significant BCVA improvement versus anti-VEGF in DME with ERM (OR = 5.287; 95% CI, 1.100–25.417), whereas no significant difference was found in eyes without ERM (OR = 1.128; 95% CI, 0.232–5.493).

**Conclusions:**

DEX therapy achieved superior short-term anatomical and functional outcomes compared to anti-VEGF in DME with ERM, supporting its consideration as a first-line treatment option in this subgroup.

**Supplementary Information:**

The online version contains supplementary material available at 10.1186/s40942-025-00788-w.

## Introduction

Diabetic macular edema (DME) is the leading cause of vision impairment in patients with diabetes. A meta-analysis reported that approximately 5.5% of patients with diabetes have DME diagnosed based on optical coherence tomography (OCT) [1]. The pathomechanism of DME involves angiogenesis and inflammation, resulting in disruption of the blood-retinal barrier [2]. Currently, anti-VEGF therapy constitutes the standard management for DME. However, approximately 40% of cases were refractory to anti-VEGF monotherapy, as indicated by persistent central macular thickness (CMT) despite treatment in Diabetic Retinopathy Clinical Research Network’s (DRCR.net) clinical trials [3]. Several studies suggested that some OCT biomarkers are associated with poor response to anti-VEGF in patients with DME, including serous retinal detachment (SRD), hyperreflective retinal foci (HRF), and disorganization of the inner retinal layers, and that steroid treatment yields better outcomes [4–6]. Unlike anti-VEGF agents, which primarily target angiogenesis, dexamethasone (DEX) 0.7 mg intravitreal implants (Ozurdex, Allergan, Inc., Irvine, CA) exert potent anti-inflammatory effects by suppressing numerous cytokines. Inflammation is known to play a key role in the pathogenesis of DME, especially in eyes unresponsive to anti-VEGF treatment [7]. The efficacy of DEX implant for DME has been demonstrated via improvement in visual acuity (VA) and decrease in retinal thickness, even in patients with DME refractory to anti-VEGF treatments [4, 7–13].

Diabetic retinopathy (DR) and its severity are positively associated with secondary epiretinal membrane (ERM) [14]. The prevalence rate of ERM in patients with DR is reportedly 33.3% [15]. Despite this, cases of ERM have been excluded from major clinical trials, even though our previous study found that DME is associated with ERM in 20.8% of patients [16]. Previous real-world and meta-analysis studies also indicated that ERM is a significant predictor of poor response to anti-VEGF therapy for DME [16, 17], concurrent with other studies that reported that vitreomacular interface abnormalities may reduce the effectiveness of intravitreal anti-VEGF treatment in eyes with DME [18, 19]. A few studies have examined treatment outcomes in DME with coexisting ERM. However, most were limited by small sample sizes or did not assess other OCT biomarkers that may influence therapeutic response [20, 21]. Currently, consensus and knowledge about the treatment of center-involving DME with concomitant ERM are lacking. Therefore, our study extends previous comparative work by incorporating multiple OCT biomarkers and applying generalized estimating equation (GEE) modeling to account for inter-eye correlation, offering a more robust evaluation than earlier studies by Erden et al. [20] and Cakir et al. [21], while directly comparing short-term outcomes of DEX versus anti-VEGF therapy in patients with and without ERM.

## Materials and methods

### Study design and setting

**Fig. 1:**
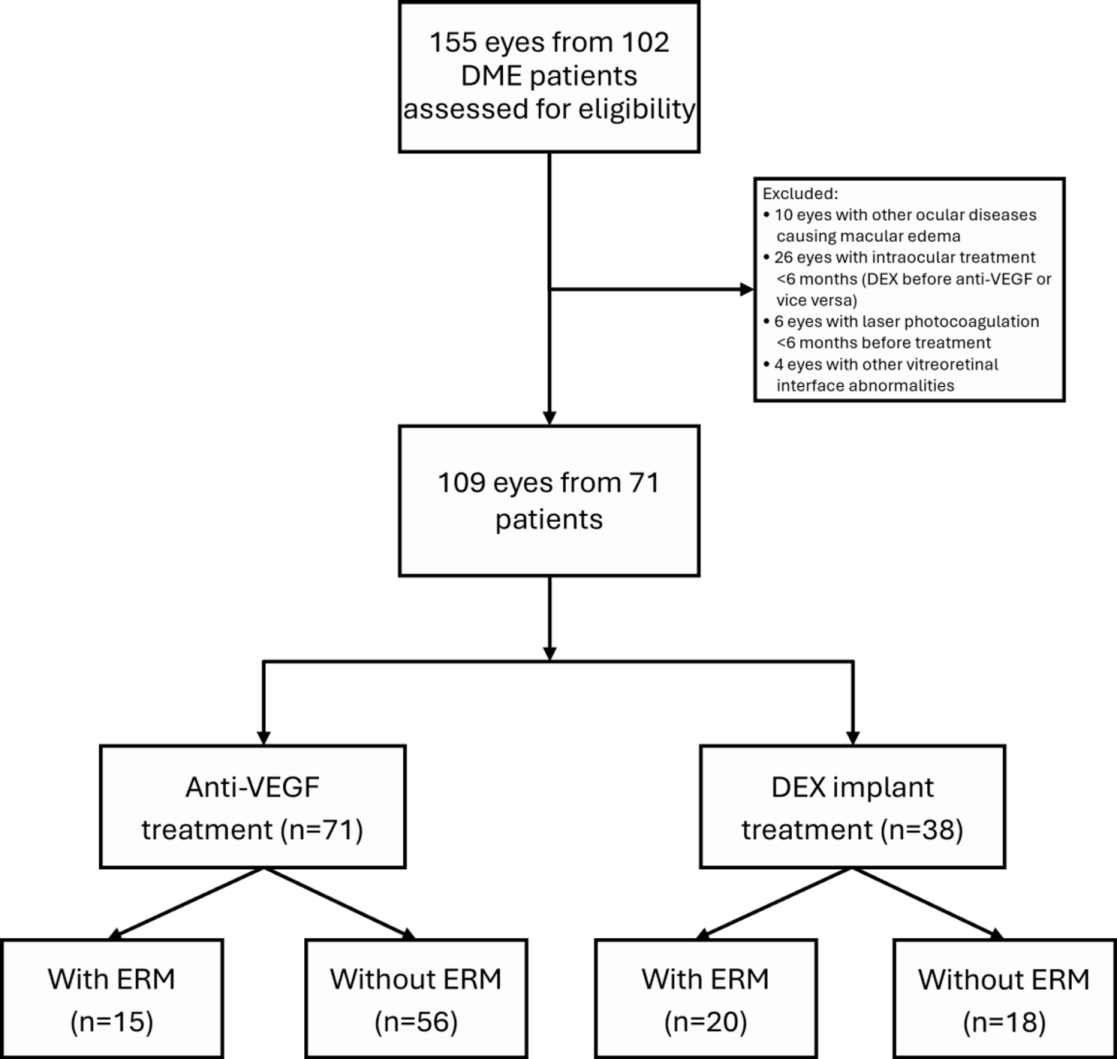
Flowchart of study participants for inclusion and treatment grouping in the DME cohort. Of 155 eyes assessed, 109 were included and analyzed by ERM status and treatment type. DEX: dexamethasone. DME: diabetic macular edema. ERM: epiretinal membrane. VEGF: vascular endothelial growth factor

This retrospective observational study was conducted at Hualien Tzu-Chi Hospital between January 1, 2014, and December 31, 2022. The study protocol was approved by the Institutional Review Board of the Research Ethics Committee of Hualien Tzu-Chi Hospital and Buddhist Tzu-Chi Medical Foundation (IRB113-033-B) and adhered to the tenets of the Declaration of Helsinki. Given the retrospective nature of the study and the use of anonymized data, the requirement for informed consent was waived by the IRB. Treatment assignment (anti-VEGF vs. DEX) was not randomized but determined by the treating physician after discussion with the patient, considering clinical factors (e.g., systemic status, visit adherence) and patient preference. No institutional guideline mandated the use of one treatment over another during the study period. Although propensity score matching was not used, baseline differences between groups were adjusted for in the multivariable analysis. The flowchart of the study design is shown in Fig. 1.

## Study participants

Eligible participants met the following criteria: (1) aged 20 years or older; (2) diagnosed with type 1 or type 2 diabetes mellitus; (3) presence of DME with associated vision impairment, defined clinically and confirmed by central subfield thickness exceeding 300 μm and evidence of intraretinal or subretinal fluid on spectral-domain OCT; and (4) received either a single intravitreal DEX implant or three-monthly injections of anti-VEGF agents (bevacizumab, ranibizumab, or aflibercept). Exclusion criteria included: (1) coexisting ocular conditions known to cause macular edema, such as neovascular age-related macular degeneration, choroidal neovascularization from other causes, retinal vein occlusion, uveitis, or recent intraocular surgery (e.g., cataract extraction or vitrectomy) that could influence drug efficacy or cause postsurgical macular edema; (2) prior intraocular corticosteroid use within 6 months before anti-VEGF therapy or anti-VEGF treatment within 6 months before DEX administration; (3) receipt of laser photocoagulation within the 6 months preceding either treatment; and (4) presence of other vitreomacular interface disorders, including vitreomacular adhesion or traction. When both eyes met inclusion criteria and received treatment, both were included in the analysis. Patients were monitored monthly over a 4-month follow-up period with comprehensive ophthalmic assessments at each visit, including best-corrected visual acuity (BCVA), intraocular pressure measurement, slit-lamp biomicroscopy, OCT imaging, and indirect ophthalmoscopy. There was no formal sample size calculation in the current study. All the eligible patients who met the enrollment criteria were enrolled for analysis between January 1, 2014, and December 31, 2022.

**Fig. 2:**
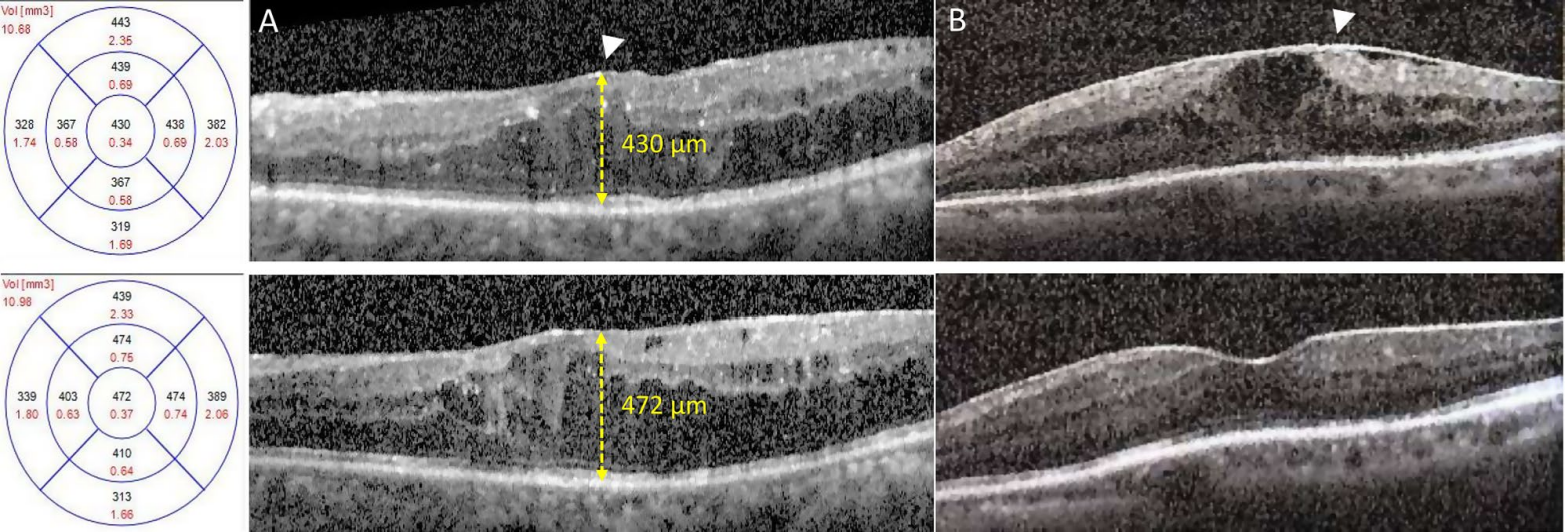
Representative OCT images of eyes with DME and ERM treated with anti-VEGF or DEX implant. (**A**) Upper: Baseline OCT shows DME with ERM (arrowhead); the eye was treated with ranibizumab (baseline CMT: 430 μm). Lower: After three consecutive monthly injections, the DME persisted with increased CMT (472 μm) at the 4-month follow-up, indicating a poor response. (**B**) Upper: Baseline OCT of another eye with DME and ERM, treated with DEX. Lower: At the 2-month follow-up, OCT shows marked regression of DME with reduction in retinal thickening, demonstrating a favorable anatomical response to DEX. CMT: central macular thickness; DME: diabetic macular edema; DEX: dexamethasone implant; ERM: epiretinal membrane; VEGF: vascular endothelial growth factor

According to a pharmacokinetics study, DEX concentrations reached maximum levels in the retina (C max = 1110 ± 284 ng/g) and vitreous (C max = 213 ± 49 ng/mL) on day 60 [22]. Additionally, the results of post hoc analysis of the DRCR.net Protocol I study suggest that the early CMT response at week 12 after 3-monthly anti-VEGF injections is predictive of the long-term anatomical outcomes of DME [23]. Therefore, our primary aim was to compare CMT relative reduction at 2 months post-DEX implant placement versus 4 months after anti-VEGF injection (Fig. 2), with the secondary goal of comparing the > 5 ETDRS letters gain in BCVA between DEX implantation and anti-VEGF treatment.

## OCT analysis

All eyes were imaged using spectral-domain optical coherence tomography (SD-OCT; Heidelberg Spectralis, Heidelberg, Germany). At baseline, both qualitative and quantitative assessments of the fovea-centered SD-OCT scans were conducted to identify key morphological characteristics. These included: (1) the presence of an ERM; (2) the presence and extent of diffuse retinal thickening (DRT), categorized by width (≤ 1 mm, 1–3 mm, or 3–6 mm); (3) cystoid changes within the outer nuclear layer, with maximum cyst diameter classified as small (< 100 μm), large (100–200 μm), or giant (> 200 μm); (4) HRF, evaluated by number (few: 2–10; moderate: 11–20; many: >20) and distribution (inner retina, outer retina, or throughout all layers); (5) presence of SRD; (6) the severity of diabetic retinopathy; and (7) CMT. The features mentioned above were evaluated using a horizontal B-scan encompassing the fovea. Two experienced retina specialists (MS He and YC Chang), blinded to the outcome, evaluated the OCT images and reached a consensus on the presence of morphological features. CMT was recorded at baseline and 1, 2, 3, and 4 months after treatment.

### Statistical analysis

All statistical analyses were conducted using SPSS for Windows (version 21.0; IBM, Armonk, NY, USA). Quantitative variables were expressed as the mean with standard deviation, while qualitative variables were presented as frequencies with percentages. GEE was employed to estimate population-averaged effects of DEX implants and anti-VEGF agents for DME concomitant with ERM, accounting for within-cluster correlation (two eyes from the same patient). Compared to mixed effects models, GEE minimizes reliance on assumptions about random effects distributions. Therefore, it offers a methodologically appropriate framework for analyzing clustered data in this context. Two statistical models were employed to address the study objectives. An exchangeable working correlation structure was specified, and robust (Huber–White sandwich) standard errors were applied to obtain valid inferences even under potential misspecification of the correlation structure. Model fit was evaluated by comparing quasi-likelihood under the independence model criterion (QIC) across candidate models, with lower QIC values indicating better model fit.

In the first model, the generalized estimate equation incorporated an interaction term between the treatment groups (DEX versus anti-VEGF) and ERM based on data from all participants. This model aimed to assess whether the effect of DEX, compared with anti-VEGF, differed between patients with and without ERM, adjusting for other baseline characteristics, including gender, baseline of CMT, baseline of BCVA, diffuse retinal thickening, ONL cyst size, HRF foci quantity, HRF foci location, SRD, and DR type. The presence of a statistically significant interaction effect would indicate substantial differences in the impact of DEX between patients with and without ERM. In the second model, subgroup analysis was performed without the interaction mentioned above to evaluate the effect of DEX compared with anti-VEGF separately for patients with and without ERM. For the comparison of demographic and baseline characteristics of the patients between the DEX and Anti-VEGF groups, GEE was also employed accounting for the cluster effect of the both eyes from same patients. A p-values were two-sided, and statistical significance was set at *p* < 0.05.

## Results

### Study participants and anatomic baseline characteristics

The analysis included a total of 109 eyes of 71 patients. The patients’ demographic characteristics are shown in Table 1. All eyes with DME were treated with either a single dose of DEX implants (*n* = 38, 34.9%) or three consecutive monthly intravitreal injections of anti-VEGF (*n* = 71, 65.1%). Three types of anti-VEGF drugs were used in our cohort, the most common of which was ranibizumab (*n* = 60 eyes, 84.5%), followed by aflibercept (*n* = 9, 12.7%) and bevacizumab (*n* = 2, 2.8%), respectively. These agents were grouped together as anti-VEGF agents for analysis, given the limited sample size per drug and their reported similar efficacy in treating DME. According to the SD-OCT images, ERM was present in 35 eyes (32.1%) and absent in 74 eyes (67.9%). OCT review indicated that ERM was already present during prior DME treatment in previously treated eyes. Among the ERM group, 20 eyes (57.1%) received DEX implants and 15 eyes (42.9%) were treated with anti-VEGF. In the non-ERM group, 18 eyes (24.3%) received DEX and 56 eyes (75.7%) received anti-VEGF therapy. Furthermore, 48 eyes (44.0%) had proliferative diabetic retinopathy (PDR), 54 eyes (49.5%) had severe non-proliferative diabetic retinopathy (NPDR), and 7 eyes (6.4%) had moderate NPDR. Based on baseline OCT findings, DRT (78%) and HRF (88.1%) were the most frequently observed morphological biomarkers.

**Table 1:** Descriptive statistics: demographic data and optical coherence tomography baseline measures

	**Variables are based on number of subjects**				
**Sex**			N (%)		
Female			25 (35.2%)		
Male			46 (64.8%)		
**Age (yrs)**,** Mean (SD)**			63.5(9.5)		
	**Variables are based on treatment for eyes**				
**Baseline Measures**		**All patients** ***N*** **= 109**	**DEX** ***N*** **= 38**	**Anti-VEGF** ***N*** **= 71**	***p*** **-value**
**ERM**					0.001
Yes		35 (32.1%)	20 (52.6%)	15 (21.1%)	
No		74 (67.9%)	18 (47.4%)	56 (78.9%)	
**Diffuse retinal thickening**					0.559
3–6 mm		49 (45.0%)	17 (44.7%)	32 (45.1%)	
1–3 mm		31 (28.4%)	9 (23.7%)	22 (31.0%)	
≤1 mm		5 (4.6%)	1 (2.6%)	4 (5.6%)	
0 mm		24 (22.0%)	11 (28.9%)	13 (18.3%)	
**ONL cyst size**					0.840
Giant		6 (5.5%)	3 (7.9%)	3 (4.2%)	
Large		29 (26.6%)	11 (28.9%)	18 (25.4%)	
Small		17 (15.6%)	6 (15.8%)	11 (15.5%)	
No		57 (52.3%)	18 (47.4%)	39 (54.9%)	
**HRF foci-quantity**					0.150
Many (> 20)		17 (15.6%)	9 (23.7%)	8 (11.3%)	
Moderate (11–20)		31 (28.4%)	11 (28.9%)	20 (28.2%)	
Few (2–10)		48 (44.0%)	15 (39.5%)	33 (46.5%)	
Absent		13 (11.9%)	3 (7.9%)	10 (14.0%)	
**HRF foci-location**					0.501
ILM-INL		28 (25.7%)	8 (21.1%)	20 (28.2%)	
OPL-ONL		10 (9.2%)	5 (13.2%)	5 (7.0%)	
Both layers		58 (53.2%)	22 (57.9%)	36 (50.7%)	
No		13 (11.9%)	3 (7.8%)	10 (14.1%)	
**SRD**					0.410
Yes		27 (24.8%)	11 (28.9%)	16 (22.5%)	
No		82 (75.2%)	27 (71.1%)	55 (77.5%)	
**DR type**					0.010
PDR		48 (44.0%)	24 (63.2%)	24 (33.8%)	
Severe NPDR		54 (49.5%)	12 (31.6%)	42 (59.2%)	
Moderate NPDR		7 (6.4%)	2 (5.3%)	5 (7.0%)	
**Baseline BVCA(Snellen decimal)**,** Mean (SD)**		0.238(0.204)	0.251(0.24)	0.23(0.19)	0.715
**Baseline CMT(µm)**,** Mean (SD)**		473.310(147.436)	444.701(167.012)	481.412(129.325)	0.205

BCVA: best corrected visual acuity; CMT: central macular thickness; DEX: dexamethasone implants; DR: diabetic retinopathy; ELM: external limiting membrane; ERM: epiretinal membrane; HRF: hyperreflective foci; ILM: internal limiting membrane; INL: inner nuclear layer; NPDR: non-proliferative diabetic retinopathy; ONL: outer nuclear layer; OPL: outer plexiform layer; PDR: proliferative diabetic retinopathy. SD: standard Deviation; SRD: serous retinal detachment; VEGF: vascular endothelial growth factor

### Reduction (improvement) in CMT after DEX implants vs. anti-VEGF

We compared CMT reduction between a single-dose DEX implant (assessed at 2 months) and three consecutive monthly anti-VEGF injections (assessed at 4 months) in patients with DME, stratified by the presence or absence of ERM. The results are summarized as follows:

### CMT reduction – DME with ERM

**Fig. 3:**
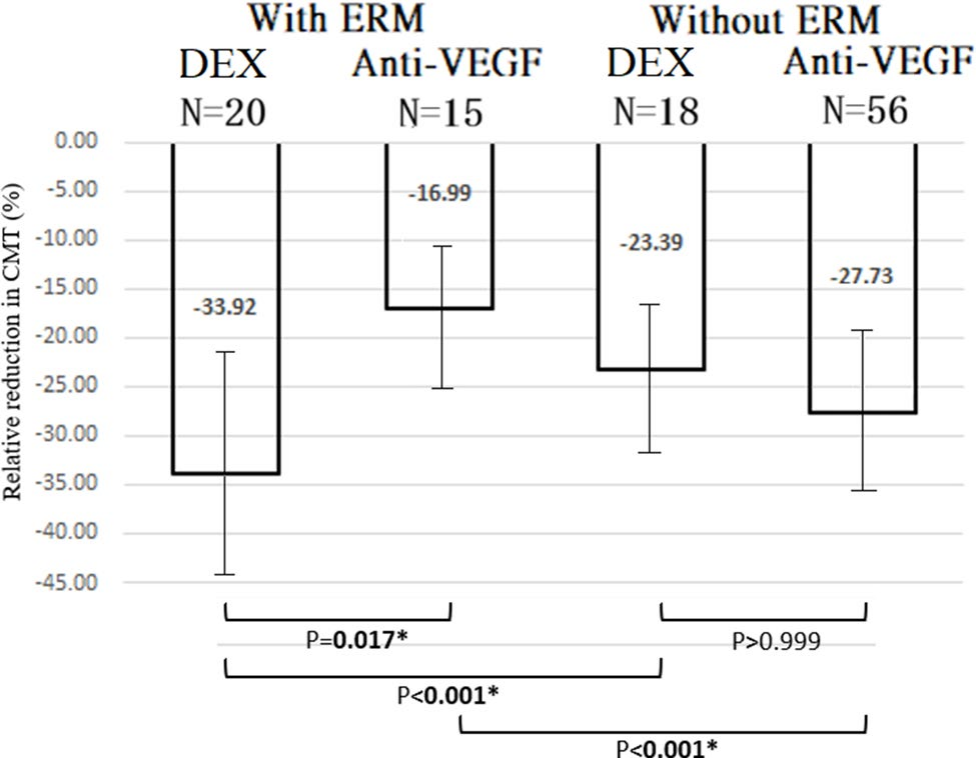
Model-adjusted mean relative reduction (improvement) in CMT after treatment with DEX vs. anti-VEGF. P-values were calculated using Bonferroni’s post-hoc test. *p-value < 0.05. CMT: central macular thickness; DEX: dexamethasone implant; ERM: epiretinal membrane; VEGF: vascular endothelial growth factor

DEX implants resulted in significantly greater CMT reduction than anti-VEGF therapy (β = − 15.675%; Wald chi-square = 9.707; *P* = 0.002). The model-adjusted mean relative CMT reduction was also significantly greater with DEX (*P* = 0.017). DEX was more effective in patients with ERM than those without (*P* < 0.001), whereas anti-VEGF was significantly less effective in the presence of ERM (*P* < 0.001) (Fig. 3).

**Fig. 4:**
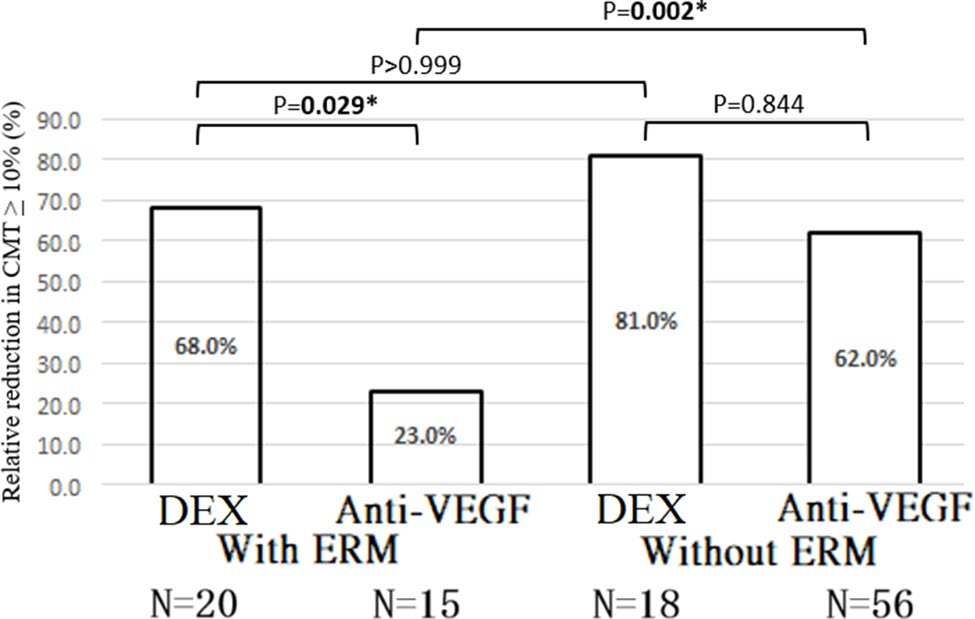
Model-adjusted proportion with relative reduction (improvement) in CMT *≥* 10% after treatment with DEX vs. anti-VEGF. P-values were calculated using Bonferroni’s post-hoc test. *p-value < 0.05. CMT: central macular thickness; DEX: dexamethasone implant; ERM: epiretinal membrane; VEGF: vascular endothelial growth factor

The proportion of eyes achieving a ≥ 10% CMT reduction was significantly higher in the DEX group [OR = 4.569; 95% CI = (4.059, 5.143); Wald chi-square = 632.304; *P* < 0.001] (Table 2). Model-adjusted proportions also favored DEX over anti-VEGF in this subgroup (*P* = 0.029) (Fig. 4).

**Table 2:** Comparison of reduction (improvement) of CMT after treatment with DEX vs. anti-VEGF (reference group: Anti-VEGF)

Subgroup	Relative reduction (improvement) of CMT						Relative reduction (improvement) of CMT *≥* 10%				
	Post-treatment of CMT(µm)		β	SE	Wald Chi-Square	P-value	Percent of CMT *≥* 10%		OR(95% CI)	Wald Chi-Square	P-value
	DEX Means (SD)	Ant-VEGF Means (SD)					DEX n (%)	Ant-VEGF n (%)			
With ERM	382.625 (61.447)	452.733 (83.275)	-15.675	5.0313	9.707	0.002	14 (70.0%)	4 (26.7%)	4.569(4.059, 5.143)	632.304	< 0.001
Without ERM	398.909 (178.951)	349.411 (105.615)	4.257	8.3402	0.261	0.610	15 (83.3%)	36 (64.3%)	3.713(0.773, 17.882)	2.686	0.101
P-value (Treatment-by-ERM interaction)	< 0.001						0.380				

CMT: central macular thickness; DEX: dexamethasone implant; ERM: epiretinal membrane; OR: odds ratio; SE: standard error

### CMT Reduction – DME without ERM

No significant difference in CMT reduction was observed between DEX and anti-VEGF (β = 4.257%; Wald chi-square = 0.261; *P* = 0.610) (Table 2). The model-adjusted mean relative CMT reduction was also nonsignificant (*P* > 0.999). The proportion of eyes achieving a ≥ 10% CMT reduction was similar between groups [OR = 3.713; 95% CI = (0.773, 17.882); Wald chi-square = 2.686; *P* = 0.101] (Table 2), with no significant difference in adjusted proportions (*P* = 0.844) (Fig. 4).

### Interaction effects – CMT

The treatment-by-ERM interaction for model-adjusted relative CMT reduction was statistically significant (*P* < 0.001), indicating that treatment efficacy varied based on ERM status. However, the interaction for achieving a ≥ 10% CMT reduction was not statistically significant (*P* = 0.380) (Table 2). In patients with ERM, the model-adjusted proportion achieving ≥ 10% CMT reduction was significantly higher in the DEX group (*P* = 0.029), while the difference was nonsignificant in patients without ERM (*P* = 0.844). No significant difference was observed between ERM and non-ERM subgroups for DEX (*P* > 0.999), but anti-VEGF was significantly less effective in eyes with ERM (*P* = 0.002) (Fig. 4).

**Fig. 5:**
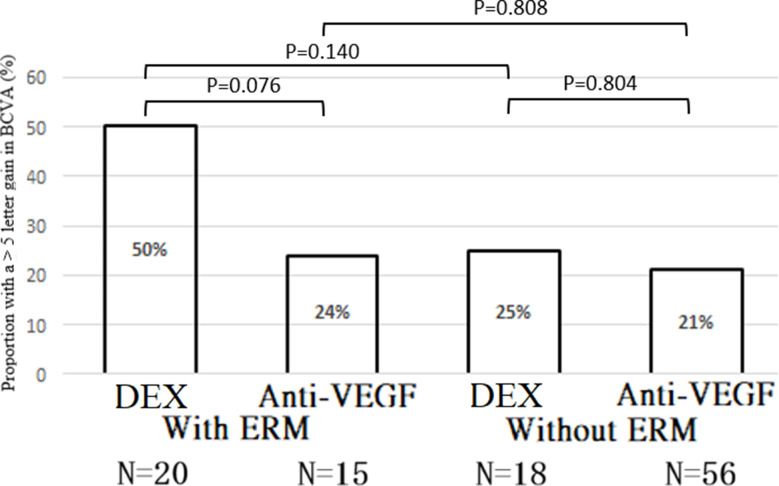
Model-adjusted proportion with a > 5-letter gain in best-corrected visual acuity after treatment with DEX vs. anti-VEGF. P-values were calculated using Bonferroni’s post-hoc test. *p-value < 0.05. DEX: dexamethasone implant; ERM: epiretinal membrane; VEGF: vascular endothelial growth factor

Additionally, we compared the achievement of a > 5-letter gain in BCVA following treatment with a single-dose DEX implant and three-monthly anti-VEGF injections in DME patients with and without ERM. In DME patients with ERM, DEX implants showed a significant benefit over anti-VEGF for a > 5-letter gain in BCVA [OR = 5.287; 95% CI = (1.100, 25.417); Wald chi-square = 4.320; *P* = 0.038], but there was no significant difference in DME patients without ERM [OR = 1.128; 95% CI = (0.232, 5.493); Wald chi-square = 0.022; *P* = 0.882]. The treatment-by-ERM interaction for a > 5-letter gain in BCVA was not significant (*P* = 0.371) (Table 3). The model-adjusted proportions of > 5-letter gain in BCVA were similar between DEX implants and anti-VEGF in both DME with ERM (*P* = 0.076) and without ERM (*P* = 0.804). No significant differences were observed between DME patients with and without ERM for either DEX implants (*P* = 0.140) or anti-VEGF treatment (*P* = 0.808) (Fig. 5).

**Table 3:** Comparison of a > 5-letter gain in best-corrected visual acuity after treatment with DEX vs. anti-VEGF (reference group: Anti-VEGF)

Subgroup	Post-treatment of BVCA		Percent of a > 5 of letter gain		OR(95% CI)	Wald Chi-Square	*P*-value
	DEX Means(SD)	Ant-VEGF Means(SD)	DEX *n*(%)	Ant-VEGF *n*(%)			
With ERM	0.158(0.107)	0.179(0.150)	11 (55%)	3(20%)	5.287(1.100, 25.417)	4.320	0.038
Without ERM	0.269(0.250)	0.307(0.222)	3(16.6%)	13(23.2%)	1.128(0.232, 5.493)	0.022	0.882
P-value (Treatment-by-ERM interaction)	0.371						

CI: confidence interval; DEX: dexamethasone implant; ERM: epiretinal membrane; OR: odds ratio; VEGF: vascular endothelial growth factor

### Safety outcomes

Intraocular pressure (IOP) elevation above 21 mmHg was observed in 6 eyes (8.5%) in the anti-VEGF group and 5 eyes (13.2%) in the DEX group during the 4-month follow-up. All cases were managed with topical medications, and no patient required surgical intervention. No eye underwent cataract surgery in either group during this period.

## Discussion

In this real-world study, we found that DEX implants yielded superior treatment outcomes compared to anti-VEGF, specifically in terms of CMT reduction and a > 5-letter gain in BCVA for DME patients with ERM compared to those without ERM. Although DEX yielded a statistically significant > 5-letter gain in ERM eyes, absolute visual improvement appears modest (≈ 0.16 logMAR); this level of gain is generally regarded as clinically meaningful in DME, especially given the limited visual potential in eyes with ERM. Furthermore, DEX implants yielded a significantly greater reduction of CMT in DME with ERM compared to DME without ERM, while anti-VEGF had the opposite effect. One notable limitation of this study is the relatively short follow-up period of 4 months, which may not fully capture the long-term treatment durability. Therefore, the findings should be interpreted with caution when considering sustained efficacy beyond this timeframe. Additionally, the different assessment timepoints (DEX at 2 months vs. anti-VEGF at 4 months) may introduce potential time-point confounding, meaning that some observed differences may reflect timing rather than true treatment superiority. Although prior DME treatments were administered more than six months before enrollment, the possibility that previous therapy reflects underlying disease severity and could influence treatment response cannot be excluded. Both treatments were generally well tolerated, though safety profiles differed slightly. Mild IOP elevations occurred in both groups, slightly more in the DEX group, consistent with known steroid effects, and were managed with topical therapy. No cataract surgeries occurred within the 4-month follow-up period; however, this timeframe may be too short to detect delayed steroid-related events, such as cataract progression or sustained IOP elevation. Therefore, the absence of severe complications should be interpreted with caution, and longer follow-up is needed to fully assess DEX safety.

Since the pathophysiology of DME involves several factors and cytokines, anti-VEGF agents are generally considered first-line management for DME. However, DME refractory to anti-VEGF injections is not a rare occurrence, according to previous studies. A post-hoc analysis of the DRCR.net Protocol I reported that approximately 40% of eyes with ranibizumab had persistent DME at the 2-year visit [23]. In the RISE/RIDE Phase III trials of ranibizumab, approximately 23% of patients had persistent DME at the 2-year follow-up [24]. The actual prevalence of refractory DME in real-world settings may be higher than estimated in these studies, as the strict enrollment and follow-up protocols used in clinical trials are unlikely to be fully replicated in standard clinical practice [25]. A key concern is the potential for early identification of patients who are unlikely to benefit from first-line anti-VEGF therapy.

ERM can develop either idiopathically or secondary to certain diseases, leading to inflammatory infiltration and cell migration from other retinal layers during its pathogenesis [26]. DR is positively associated with secondary ERM [14]. The current study found that the incidence of ERM was 32.1% in the DME cohort. Our previous study demonstrated that ERM is a significant predictor of poor response to anti-VEGF therapy in DME eyes [16]. In a retrospective study, Ercalik et al. evaluated 56 eyes with and without ERM and found that the presence of ERM negatively impacted the response to intravitreal anti-VEGF treatment [27]. Additionally, Wong et al. conducted a prospective study involving 104 eyes with DME treated with ranibizumab and concluded that the presence of ERM was associated with a poorer visual and anatomical response [28]. In contrast, a recent study demonstrated that the presence of an ERM did not alter the treatment response to DEX implants [20]. Comparative evidence on the therapeutic effects of DEX implants and anti-VEGF agents for DME with concurrent ERM is limited. Only one small retrospective study, which included 39 eyes from 34 participants, compared the effects of ranibizumab and DEX implant in DME with coexisting ERM; the results showed that both ranibizumab and DEX are effective in improving the anatomical and visual outcomes in DME with concurrent ERM [21]. Notably, that study did not evaluate other OCT biomarkers, which may bias the effect of ERM in eyes with DME after treatment.

Considering the diversity of OCT morphology in DME, we included various OCT biomarkers and considered ERM as a variable in the current analysis. We found that DEX implants yielded significantly greater therapeutic improvement in the functional and anatomical outcomes in DME with ERM compared to DME without ERM, compared with anti-VEGF. To date, consensus regarding the treatment of center-involving DME with coexisting ERM is lacking. Vitrectomy could be a relevant treatment for refractory DME with ERM because the removal of the ERM can relieve vitreomacular traction and eliminate VEGF and pro-inflammatory cytokines associated with ERM [29]. Recent studies have investigated the efficacy of vitrectomy in DME patients with coexisting ERM. Some reports have shown that these patients may experience significant improvements in VA and macular thickness following vitrectomy combined with ERM peeling [30, 31]. However, ERM secondary to DR differs from idiopathic ERM. The diabetic macula is more vulnerable to peeling-induced damage. In a prospective interventional study involving 42 eyes, Romano et al. found that surgical outcomes were less favorable in these cases, likely due to increased susceptibility to surgical trauma during membrane peeling [32].

ERM formation in diabetic eyes involves inflammatory and fibrotic pathways, including elevated TGF-β, IL-6, IL-8, and NF-κB activity [33, 34]. DEX can suppress these mediators, providing anti-inflammatory and antifibrotic effects that may explain the stronger response observed in DME with ERM [34–38], where VEGF-independent mechanisms play a larger role. Mechanically, ERM progression leads to the accumulation of contractile myofibroblasts [39], which increases macular traction and creates spaces that retain fluid, thereby sustaining DME [40]. ERM tissue also expresses factors such as VEGF, PDGF, FGF, and IL-6, which further contribute to edema through non-VEGF pathways [41]. These structural and biochemical features may reduce the effectiveness of anti-VEGF therapy by limiting drug penetration [42], promoting profibrotic signaling (e.g., CTGF) [43, 44], and driving edema via inflammatory pathways. Together, these mechanisms can result in a diminished or paradoxical response to anti-VEGF agents in eyes with ERM. Our results support this finding, revealing that anti-VEGF treatment elicits a better response in DME without ERM than in DME with ERM. Our findings contribute to the growing body of evidence on anti-VEGF-resistant DME, particularly in eyes with coexisting ERM. Previous studies have shown that structural alterations, including ERM and vitreomacular interface abnormalities, are associated with suboptimal responses to anti-VEGF therapy [42]. Real-world data also indicate that a significant proportion of DME patients exhibit incomplete anatomical or functional response after multiple anti-VEGF injections [13, 16, 27, 45]. The superior short-term effect of dexamethasone implants observed in our study suggests that early switching to corticosteroid therapy may be beneficial in selected patients with ERM-related treatment resistance. This supports a more personalized approach to DME management, emphasizing the importance of baseline imaging to guide initial treatment decisions and monitor treatment response. Despite the superior short-term effect of DEX implants, their therapeutic duration is typically limited to 3–4 months, often requiring repeated injections for sustained control. Repeated corticosteroid use carries additional safety considerations, such as intraocular pressure elevation and cataract progression [8, 46–48].

Our study has certain limitations. First, the retrospective, non-randomized design introduces selection bias, as treatment decisions were based on physician judgment and patient preference. The DEX group had more eyes with ERM and PDR, creating a risk of confounding by indication. Because the study relied on preexisting medical records, unmeasured differences—such as retinopathy severity—may have influenced outcomes. Although GEE models adjusted for measured factors, residual confounding cannot be ruled out. Second, the anti-VEGF group contained three agents, with ranibizumab used in most eyes and bevacizumab/aflibercept in only a few. Although pooled to avoid underpowered subgroup analyses, this heterogeneity may introduce bias, as the efficacy of anti-VEGF agents and ERM-related effects can differ among them. Newer anti-VEGF therapies were also not included. Therefore, caution is needed when generalizing our findings to the broader anti-VEGF class or to newer agents. To address potential drug heterogeneity, we conducted a sensitivity analysis comparing DEX with ranibizumab alone, the predominant anti-VEGF agent in our cohort; the results (Tables S1 and S2) were consistent with the primary analysis, supporting the robustness of our findings. Third, although most participants (93.6%) had severe NPDR or PDR, macular edema can also be initiated by ERM alone. Because ERM was assessed only at baseline, we cannot determine whether it contributed to the development of edema or represented a secondary change in these diabetic eyes—a fundamental pathophysiological question that our data cannot resolve. Fourth, the follow-up period was short, which may not fully represent the differences in the long-term treatment effect between DEX and anti-VEGF agents. However, among patients with DME refractory to anti-VEGF therapy after a loading dose of three consecutive monthly injections, those who were switched to other treatment modalities (e.g., corticosteroids) exhibited better visual and anatomical outcomes at 12 months than did those who continued with anti-VEGF therapy [49]. Accordingly, the early identification of patients who would not benefit from this first-line treatment is critical. Fifth, although DEX demonstrated significantly greater BCVA improvement than anti-VEGF in DME patients with ERM, this benefit was not observed in those without ERM. However, the nonsignificant treatment-by-ERM interaction (*P* = 0.371) indicates that the difference in treatment effect across ERM subgroups was not statistically meaningful. This may be partly due to variability in patient responses, as reflected by the wide confidence intervals in both groups. Additionally, anatomical improvement in CMT does not always correlate with functional gains in visual acuity. This aligns with a post hoc analysis of the Protocol T trial, which found that changes in CMT accounted for only a small portion of the variability in BCVA outcomes and concluded that CMT reduction may not fully translate into improvements in visual acuity when assessing therapeutic response in DME [50]. Sixth, due to the limited sample size, we did not perform group matching based on any specific factors. Instead, we used statistical models to evaluate the treatment effects, adjusting for other baseline characteristics. In addition, there was no formal sample size calculation, which may limit the statistical power for detecting certain treatment effects or subgroup differences. Seventh, because OCT imaging lacked a standardized grading system and historical scans did not permit reliable staging, ERM severity could not be assessed and was recorded only as present or absent. This limitation may influence the interpretation of treatment comparisons. Despite these limitations, A key strength of our study is the inclusion of ERM and a range of common OCT markers in patients with DME, providing extensive data that supported timely and individualized treatment in real-world clinical practice. Our findings suggest that ERM on baseline OCT may help guide treatment decisions. Given the limited response to anti-VEGF and better outcomes with DEX, early corticosteroid use may be considered in DME patients with ERM.

## Conclusions

DEX therapy resulted in greater short-term anatomical and visual improvements than anti-VEGF treatment in eyes with DME and ERM. Although BCVA outcomes numerically favored DEX in the ERM subgroup, the ERM–treatment interaction was not statistically significant and should be interpreted as a trend rather than a definitive differential effect. ERM remained a marker of poorer anti-VEGF response, suggesting that DEX may be a reasonable alternative in these patients, but longer-term studies are needed.

## Supplementary Information

Below is the link to the electronic supplementary material.

Supplementary Material 1

## Data Availability

All data generated in this study are included in the article, and are available from the corresponding authors upon further reasonable request.
