# Supplementary material for: Dexamethasone implant vs. anti-VEGF for diabetic macular edema with epiretinal membrane: short-term outcomes
Source: Int J Retina Vitreous. 2026 Jan 2;12:20. doi: 10.1186/s40942-025-00788-w (PMC12866193; doi:10.1186/s40942-025-00788-w)
Supplement: Supplementary file 1 — Supplementary Material 1 [file 40942_2025_788_MOESM1_ESM.docx]

Table S-1. Comparison of reduction (improvement) of CMT after treatment with DEX vs. Ranibizumab (reference group: Ranibizumab)

| Subgroup | Relative reduction (improvement) of CMT | | | | | | Relative reduction (improvement) of CMT > 10 % | | | | |
| --- | --- | --- | --- | --- | --- | --- | --- | --- | --- | --- | --- |
|  | Post-treatment of CMT(μm) | | β | SE | Wald Chi-Square | P-value | Percent of CMT > 10 % | | OR(95% CI) | Wald Chi-Square | P-value |
|  | DEX  Means  (SD) | Ranibizumab  Means  (SD) |  |  |  |  | DEX  n  (%) | Ranibizumab n  (%) |  |  |  |
| With ERM | 382.625  (61.447) | 441.912  (94.618) | -19.638 | 5.9736 | 10.808 | 0.001 | 14  (70.0%) | 2  (18.2%) | 42.693(23.025, 79.160) | 142.001 | <0.001 |
| Without ERM | 398.909  (178.951) | 351.851  (109.173) | 4.375 | 8.2398 | 0.282 | 0.595 | 15  (83.3%) | 30  (65.2%) | 3.519(0.726, 17.056) | 2.686 | 0.118 |
| P-value  (Treatment-by-ERM interaction) | <0.001 | | | | | | 0.214 | | | | |

CMT: central macular thickness; DEX: dexamethasone implant; ERM: epiretinal membrane; OR: odds ratio; SE: standard error.

Table S-2. Comparison of a > 5-letter gain in best-corrected visual acuity after treatment with DEX vs. Ranibizumab (reference group: Ranibizumab)

|  | Post-treatment of BVCA | | Percent of a > 5 of letter gain | | OR(95% CI) | Wald Chi-Square | P-value |
| --- | --- | --- | --- | --- | --- | --- | --- |
| Subgroup | DEX  Means(SD) | Ranibizumab  Means(SD) | DEX  n(%) | Ranibizumab  n(%) |  |  |  |
| With ERM | 0.158(0.107) | 0.190(0.163) | 11 (55%) | 2(18.2%) | 4.907(0.697, 24.068) | 2.436 | 0.119 |
| Without ERM | 0.269(0.250) | 0.335(0.233) | 3(16.6%) | 10(24.4%) | 1.180(0.230, 6.052) | 0.022 | 0.843 |
| P-value  (Treatment-by-ERM interaction) | 0.941 | | | | | | |

CI: confidence interval; DEX: dexamethasone implant; ERM: epiretinal membrane; OR: odds ratio; VEGF: vascular endothelial growth factor.
